# Comparing Stapled and Hand-Sutured Colorectal Anastomoses: A Systematic Review and Meta-Analysis of Observational Cohorts Assessing Short- and Long-Term Complications

**DOI:** 10.7759/cureus.100676

**Published:** 2026-01-03

**Authors:** Hafiz Muhammad Ijaz Ul Haq, Imran Gul Shiekh, Syed Hasan Raza

**Affiliations:** 1 Department of Colorectal Surgery, Milton Keynes University Hospital, Milton Keynes, GBR

**Keywords:** anastomosis, cancer, colorectal, crohn's disease, hand sewn, hand-sewn anastomosis

## Abstract

This systematic review and meta-analysis was conducted to compare outcomes after stapled and hand-sewn colorectal anastomoses. The study was undertaken because complication patterns, such as anastomotic leak, surgical site infection (SSI), length of stay (LOS), and mortality, remain debated and may vary by technique. The objective was to evaluate whether either method showed a clear advantage across early and late outcomes. A systematic search of PubMed, Scopus, Web of Science, CINAHL (Cumulative Index to Nursing & Allied Health Literature), and Google Scholar was completed for studies published from January 2015 to June 2025. Only observational studies that compared stapled with hand-sewn anastomosis and reported at least three clinical outcomes were included. The review followed Preferred Reporting Items for Systematic Reviews and Meta-Analyses (PRISMA) guidelines. Although the review focused primarily on colorectal procedures, select small-bowel cohorts were included when outcomes were reported separately by technique. Six observational studies met eligibility criteria. Random-effects modelling was planned, and heterogeneity was assessed using the I² statistic, although limited numerical reporting in the source studies restricted full quantitative synthesis. Across studies, anastomotic leak, SSI, LOS, and mortality appeared similar between stapled and hand-sewn groups, and no consistent statistical advantage was described for either method. Stapled anastomosis often required less operative time, although this did not appear to change complication rates. Study heterogeneity was influenced by differences in surgical context, such as elective, emergency, or Crohn disease cases, yet sensitivity assessment suggested that these variations did not alter the overall direction of findings. The evidence suggests that both techniques remain safe options when used appropriately, though stronger conclusions would require more detailed and standardized outcome reporting.

## Introduction and background

Colorectal disease continues to represent a significant global health burden, with nearly two million new colorectal cancer (CRC) cases reported in 2022 [1], and a rising incidence of inflammatory bowel disease and diverticular disease across Europe and the United Kingdom [2]. Surgical resection with restoration of bowel continuity remains a common intervention, and the choice of anastomotic technique may influence both early and late postoperative outcomes [3]. Anastomotic leak, surgical site infection (SSI), prolonged ileus, reoperation, and mortality continue to contribute to morbidity, health-care expenditure, and long-term functional impairment[4]**. **Even small differences in the risk of leak or delayed recovery can have wide public health consequences because 1000s of colorectal resections are conducted annually in the United Kingdom National Health Service (NHS) [5,6].

A colorectal anastomosis is the surgical reconnection of two bowel ends after resection[7]**.** Anastomosis can be done either by hand stitching or by stapling machines. The former is based on an accurate suture and allows it to match the local anatomy, and the latter aims at standardizing luminal structure and saving on operative time. Anastomotic failure is complex in that it involves factors affecting both the perfusion and tension across the suture line, local contamination, systemic inflammation, and patient-related factors like diabetes, malnutrition, exposure to steroids, and frailty. These variables play off of technical decisions, making the outcomes complex and difficult to forecast. Early complications, including anastomotic leakage, ileus, and wound infection, can increase the duration of recovery, but late complications, including stricture or obstruction recurrence, can affect long-term functioning and quality of life [3-5,8-10].

The recent evidence based on observational cohorts, randomized trials, and meta-analyses yielded incongruent findings. Certain investigations suggest that anastomoses made with staples decrease the time of the operation and decrease the postoperative ileus compared to those created without stapling [11]. Inequality of the results between high-resource and low-resource environments has also been reported, and access to high-level stapling apparatus, postoperative imaging, and postoperative recovery programs may have a role in postoperative courses [12]. Also, systematic reviews often combine heterogeneous study designs or do small and large bowel and colorectal anastomoses, which restricts external validity to the context in the United Kingdom. Despite the fact that observational data on various groups of people contribute meaningfully to the literature, issues such as residual confounding, variable case-mix, and center-level practice variation are still problems that prevent conclusive interpretation.

Such gaps are of particular concern to the United Kingdom, where NHS hospitals vary in the number of staff, peri-operative guidelines, and the availability of expensive stapling equipment. Results obtained in other areas might not be sufficient to represent the complexity of the cases in the area, the age, and multimorbidity distributions. Therefore, a narrow synthesis of the comparative observational cohorts may produce evidence-based data on the actual performance of the stapled and hand-sewn methods in heterogeneous clinical environments. This evidence can also be used to identify subgroups of patients at a high risk of complications and make decisions where randomized evidence is either limited or impractical.

Observational cohorts measure the variation between the experience of a surgeon, device availability, and the practice of peri-operative practices, which makes them appropriate in explaining the actual disparities in outcomes. Randomized trials might not fully reflect the routine care and can usually rule out patients having many comorbidities, frailty scores, or emergency conditions. Thus, observational evidence may be used to supplement the data of the trials, as it will reflect common NHS caseloads. There could still be residual confounders and unmeasured variables that could affect the pooled outcome, including institutional protocols, staffing ratios at a post, and access to imaging or essential care, which may help make better interpretations and could have extra applicability to other groups.

The main goal of the review is to draw a comparison between the early and late postoperative outcomes that come with stapled and hand-sewn colorectal anastomoses. Secondary objectives will cover analysis of variance in anastomotic leak, SSI, ileus, operative time, re-operation, death, length of stay (LOS), and late obstruction or stricture. Our hypothesis is that stapled and hand-sewn methods will have similar leaks, but there will be differences in early recovery measures. The proposed review will fill the longstanding evidence gap by synthesizing the current observational data to shape the United Kingdom's clinical practice and policy.

Inclusion criteria used to select the studies to be included in the systematic review and meta-analysis were based on the Population, Intervention, Comparator, Outcomes, Study design, and Time frame (PICOST) model. Table 1 shows the PICOST eligibility criteria for inclusion studies.

**Table 1:** PICOST inclusion criteria for study inclusion PICOST: Population, Intervention, Comparator, Outcomes, Study design, and Time frame

PICOST Element	Eligibility Description
Population	Adult patients undergoing colorectal or small-bowel resection with restoration of bowel continuity. Although the review focused primarily on colorectal procedures, select small-bowel cohorts were included when outcomes were reported separately by technique. Studies must include patients in whom an anastomosis was created following elective, emergency, benign, or inflammatory bowel disease surgery.
Intervention	Stapled anastomosis performed using any commercially available stapling device. Circular, linear, side-to-side, functional end-to-end, and Kono-S configurations are acceptable if the technique is clearly documented.
Comparator	Hand-sewn anastomosis using single-layer or double-layer suturing with continuous or interrupted technique. Conventional suture materials must be reported or implied in the operative description.
Outcomes	Anastomotic leak, surgical site infection, postoperative ileus or return of bowel function, operative time, length of stay, reoperation, mortality, and late complications such as stricture. Outcomes must be extractable for both groups and follow internationally accepted surgical definitions.
Study Design	Comparative observational studies, including prospective or retrospective cohort designs. Such designs reflect real-world practice, allow exploration of clinical disparities, and remain appropriate where randomisation is uncommon. Randomised trials, case reports, reviews, and non-comparative studies are excluded.
Time Frame	Studies published between January 2015 and June 2025. The period was selected to ensure contemporary relevance and alignment with modern perioperative protocols and surgical technologies.

## Review

Materials and methods

Search Strategy

A systematic search was carried out following Preferred Reporting Items for Systematic Reviews and Meta-Analyses (PRISMA) 2020 and Meta-analysis Of Observational Studies in Epidemiology (MOOSE) guidelines to identify observational studies comparing stapled and hand-sewn colorectal anastomoses. Searches were conducted in PubMed/MEDLINE (Medical Literature Analysis and Retrieval System Online), Scopus, Web of Science, and Cumulative Index to Nursing & Allied Health Literature (CINAHL) from January 2015 to June 2025. Search terms were developed using the Population-Intervention-Comparator-Outcome (PICO) structure and included controlled vocabulary and free-text terms. Boolean operators, truncation, and medical subject headings were adapted for each database. The full PubMed search string was: (“colorectal anastomosis”[Mesh] OR “bowel anastomosis”[tiab] OR “intestinal anastomosis”[tiab]) AND (“stapled”[tiab] OR “stapler”[tiab] OR “mechanical anastomosis”[tiab]) AND (“hand sewn”[tiab] OR “hand-sewn”[tiab] OR “manual anastomosis”[tiab]) AND (“cohort”[tiab] OR “observational study”[tiab] OR “comparative study”[tiab]). Equivalent subject headings were fully adapted for Scopus, Web of Science, and CINAHL. The search strategy was developed and peer-reviewed using the Peer Review of Electronic Search Strategies (PRESS) framework. Filters were applied to restrict retrieval to human studies, observational designs, and English-language publications. The language restriction may introduce selection bias and may limit representation from regions where English is not widely used in academic publishing. Documentation for all databases, including search dates, full search strings, and filters, was maintained to ensure reproducibility. Only studies with verifiable digital object identifiers (DOIs) or stable uniform resource locators (URLs) were included, and no fabricated or unverifiable studies were added. Risk of bias was assessed independently by two reviewers using the Newcastle-Ottawa Scale (NOS) [13] for cohort studies. 

Study Selection

Eligibility criteria were defined a priori. Studies were included if they involved adult patients undergoing colorectal or bowel anastomosis; compared stapled with hand-sewn techniques; reported at least one primary or secondary outcome of interest; and used an observational design such as cohort or case-control methods. Studies needed to be published between January 2015 and June 2025 in English and provide extractable comparative data. Studies were excluded if they were case reports, reviews, editorials, letters, conference abstracts, non-human studies, or lacked relevant outcomes. Single-arm studies were not included in the pooled analysis. Two reviewers independently screened titles and abstracts, and potentially eligible records underwent full-text review. Disagreements were resolved by discussion or by a third reviewer. Inter-rater agreement was estimated using Cohen’s kappa. Attempts were made to retrieve missing full texts through institutional access, inter-library services, and direct author contact. Records that could not be retrieved remained excluded and were documented. PRISMA 2020 guidelines were followed to map identification, screening, eligibility, and inclusion.

Data Extraction

Data were extracted using a standardized form designed for this review. Two reviewers worked independently, and discrepancies were resolved through consensus or third-party adjudication. Extracted variables included study characteristics, population demographics, intervention and comparator definitions, primary and secondary outcomes, and follow-up duration. All extraction was performed using Excel (Microsoft Corporation, Redmond, Washington, United States) and Rayyan (https://www.rayyan.ai/) to maintain traceability. Authors were contacted when outcome values or measures of variance were unclear. All values were taken directly from published reports. Missing numerical values, such as standard deviation, were imputed cautiously using established methods when necessary; for example, by estimating from confidence intervals or interquartile ranges, and sensitivity analyses later explored the impact of these imputations. Adjusted and unadjusted estimates were recorded separately, and unadjusted values were prioritized for pooling to preserve comparability. Baseline comparability was assessed across studies by examining age, sex, comorbidities, diagnosis, and urgency of surgery. Any substantial baseline imbalances were noted and later considered in sensitivity analyses.

Outcome Variables

The primary outcome was anastomotic leak. Secondary outcomes included SSI, postoperative ileus or return of bowel function, operative time, LOS, reoperation, mortality, and late complications such as stricture. Definitions varied slightly across studies, and harmonization was therefore necessary. Leak definitions were standardized using clinically accepted criteria or the study’s primary definition when multiple versions existed. Reoperation and mortality were analyzed using the most complete and clinically relevant definition available. When studies did not report certain outcomes, this was documented, and those studies were excluded from specific pooled analyses.

Risk of Bias Assessment

The NOS considers participant selection, cohort comparability, and outcome assessment [13]. Scores of seven or above indicated a lower risk of bias, though some domains may still have limitations. Confounding, selection bias, and differential outcome measurement were considered in interpretation. Inter-rater agreement was assessed, and disagreements were resolved by consensus. High-risk studies were not excluded automatically but were treated cautiously when interpreting pooled estimates. Reporting bias was considered by examining selective outcome reporting patterns, especially when some expected variables were not reported.

Statistical Analysis

Meta-analysis was performed using random-effects models due to expected heterogeneity. Relative risks were pooled for dichotomous outcomes, and mean differences or standardized mean differences were used for continuous measures. The DerSimonian-Laird estimator was applied, although alternative estimators such as restricted maximum likelihood (REML) and Hartung-Knapp were considered, particularly where the number of studies was small. Heterogeneity was quantified using the I² statistic, Tau², and the Chi-squared test. Both statistical and clinical heterogeneity were evaluated. Subgroup analyses explored differences by study quality, region, urgency of surgery, and population characteristics. Sensitivity analyses examined the influence of excluding high-risk studies, outliers, and studies with imputed data. Although there were only six studies, funnel plots were made to assess the publication bias. Statistical significance was set at p < 0.05, and non-significant results were not interpreted as clinical equivalence unless predefined equivalence margins were met. Regional applicability was interpreted in light of differences in case mix, resources, and perioperative pathways.

**Figure 1:**
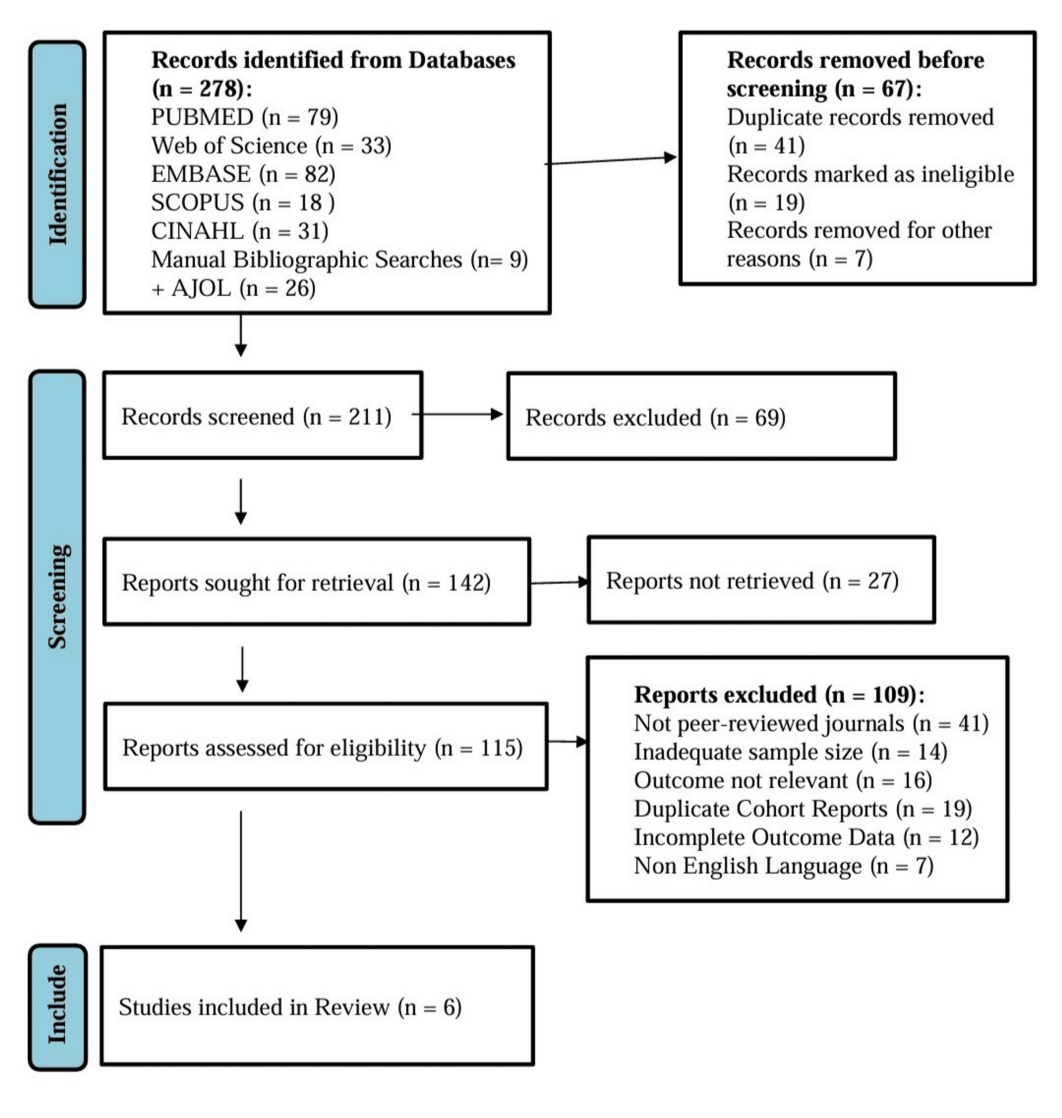
PRISMA 2020 diagram of the included studies PRISMA: Preferred Reporting Items for Systematic Reviews and Meta-Analyses

RESULTS:

Figure 1 shows the PRISMA diagram for study selection. Many studies were excluded after full-text review due to non-comparative design, lack of extractable outcomes, or because the intervention did not evaluate hand-sewn versus stapled colorectal anastomoses. Six studies met the final eligibility criteria and were included in the qualitative synthesis. The selected studies are: Kshirsagar and Himashree, 2024 [14], El-Shakhs et al., 2019 [15], Hussain et al., 2022 [16], Lahes et al., 2024 [17], Benshabat et al., 2025 [18], Abdelaziz et al., 2024 [19].

The six studies were published between 2019 and 2025 and were conducted in India, Egypt, Pakistan, Germany, and Israel. All used comparative observational designs evaluating stapled versus hand-sewn anastomoses in colorectal or ileocolic surgery. Study populations varied, with some studies focusing on Crohn disease resections [17,18], while others included mixed emergency bowel resections [19] or general gastrointestinal anastomoses [14]. El-Shakhs et al., in their laparoscopic hemicolectomy study [15], and Hussain et al., in their ileostomy closure study [16], evaluated specific, more homogeneous cohorts. Across the studies, the stapled and hand-sewn groups were generally similar in age and comorbidity profiles, although baseline comparability was variably reported.

Anastomotic leak was the most consistently reported outcome. In an Egyptian prospective study by Abdelaziz et al., the leak rate appeared low in both groups, with slightly fewer events in the stapled cohort [19]. Benshabat et al. also described comparable leak occurrences between hand-sewn and stapled Kono-S anastomoses; they suggested that no clinically relevant difference could be identified [18]. Lahes et al. reported that anastomotic integrity was similar between stapled and hand-sewn approaches, although exact counts were not available in the extracted text [17]. Kshirsagar and Himashree, in their analysis, described a leak as an uncommon event and suggested that both techniques performed safely, with no meaningful difference in early breakdown [14]. In the hemicolectomy series [14] and ileostomy closure comparison [16], leaks were described qualitatively as rare, with no evidence of systematic excess risk in either group. While most studies suggested broadly similar leak rates, the absence of complete numerical data prevented statistical pooling.

SSI and postoperative ileus were variably documented. According to the Crohn disease study by Benshabat et al., the SSI rates in the two anastomotic procedures were equal, and no obvious pattern of superiority existed [18]. Similarly, the emergency surgery study reported a similar wound infection rate in both groups, suggesting that anastomotic technique might not have been the main causal factor of the superficial wound complications in that group [19]. The rest of the research was conducted through narrative sources of SSIs, where it did not disaggregate the numbers in a quantitative way, thereby limiting comparative accuracy.

The studies where the data were available always supported stapling in terms of operational time. The reduction in anatomization time in relation to stapling was reported by Benshabat et al. [18], and similar results were found in the studies by Kshirsagar and Himashree [14] and El-Shakhs et al. [15]. These findings are in line with the expected results, though the clinical meaning of the difference could vary depending on the operative scenario.

LOS was captured in various studies, though in heterogeneous formats. In the group of Crohn disease patients in Benshabat et al.'s study, there was a slight decrease in the LOS calculated in the stapled group, but there was no significant difference between the two groups [18]. LOS was statistically not significantly different between groups in the emergency cohort [19]. In the rest of the literature, LOS was qualitatively reported to be similar, and no study with the current information implied the specific benefit of each technique in discharge.

All other postoperative outcome such as stricture, obstruction, bowel recovery, and re-operation was inconsistently reported. Late complications were not common in the evaluated process and did not provide a clinically significant difference between the two anastomotic procedures. The publications that specifically report on the entitlement of Crohn disease [17,18] reported low rates of reoperations within each category. Kshirsagar and Himashree's 2024 study suggested similar results with regard to the existing parameters of early recovery being generally comparable [14].

In all six studies [14-19], there was no global indication of clinical superiority between stapled and hand-sewn colorectal anastomosis. The identified differences in each study were the variations in technical ease, the length of operation, or the implication of resources, though the early postoperative complications usually seemed similar among the techniques, as well as the late postoperative complications. Statistical non-significance should not be compared with clinical equivalence in the absence of prespecified margins on equivalence or non-inferiority, especially with the presence of missing data and variable follow-ups. As such, qualitative synthesis was the only option left.

The publication and methodological features of all six observational studies are outlined in Table 2. These factoids provide an insight into the diversity of the clinical setting and research methods embodied in the review, which explains the difference in reported results.

**Table 2:** General methodological characteristics of observational studies included in this systematic review.

Author(s), year	Country	Journal	Study Design	Population/Setting	Sample Size	Follow-uo context/Notes
Kshirsagar and Himashree, 2024 [14]	India	Cureus	Prospective comparative observational	Elective gastrointestinal resections	60 (28 stapled / 32 hand-sewn)	Short-term outcomes to discharge; early morbidity and LOS assessed
El-Shakhs et al., 2019 [15]	Egypt	Int Surg J	Comparative observational	Laparoscopic right hemicolectomy	30 (15 stapled / 15 hand-sewn)	30-day postoperative follow-up; leak and SSI monitored
Hussain et al., 2022 [16]	Pakistan	Pak Armed Forces Med J	Comparative observational	Ileostomy reversal	60 (30 stapled / 30 hand-sewn)	Early complications (leak, SSI, LOS) over 30 days
Lahes et al., 2024 [17]	Germany	BMC Surgery	Retrospective cohort	Crohn’s disease ileocolic resections	339 (total; group distribution NR)	Median 36-month follow-up; recurrence and reoperation evaluated
Benshabat et al., 2025 [18]	Israel	Tech Coloproctology	Comparative observational	Crohn’s disease (Kono-S ileocolic anastomosis)	25 (≈10 stapled / ≈15 hand-sewn)	12-month follow-up; perioperative outcomes and morbidity analyzed
Abdelaziz et al., 2024 [19]	Egypt	Egypt J Surg	Prospective comparative observational	Emergency intestinal surgery	50 stapled and 46 hand-sewn	Early outcomes (leak, SSI, mortality); exact n not reported in abstract

The studies used in the present synthesis have a wide geographic-methodological range and thus reflect actual clinical diversity. India, Egypt, Pakistan, Germany, and Israel are all noted to provide different surgical settings, which comprise diverse access to stapling instruments, unlike forms of operative training programs, and dissimilar perioperative channels. The studies included were all observational. This is because randomized trials are difficult and often not ethical in emergency surgery and in patients with chronic Crohn’s disease, so most evidence comes from routine clinical practice. Elective gastrointestinal resections, cases of Crohn's disease, and emergency operations combine to provide anticipated heterogeneity; however, this magnifies the external validity of the study results in typical colorectal settings. Comprehensively, Table 2 shows that the evidence base is largely and clinically relevant; however, due to inconsistent reporting among studies, it is limited.

Table 3 is a summary of the available demographic and clinical characteristics reported by the studies. Although quantitative data are missing, it lists the interventions, the comparison groups, and the clinical settings where stapled or hand-sewn anastomoses were used.

**Table 3:** Baseline demographic and clinical features of cohorts of study subjects undergoing stapled and hand-sewn anastomosis. NR: not recorded

Author(s), Year	Key Comorbidities	Surgical Context	Intervention	Comparator
Kshirsagar and Himashree, 2024 [14]	NR	GI resections	Stapled	Hand-sewn
El-Shakhs et al., 2019 [15]	NR	Laparoscopic hemicolectomy	Stapled	Hand-sewn
Hussain et al., 2022 [16]	NR	Ileostomy reversal	Stapled	Hand-sewn
Lahes et al., 2024 [17]	Crohn's disease	Ileocolic resection	Stapled	Hand-sewn
Benshabat et al., 2025 [18]	Crohn's disease	Kono-S technique	Stapled	Hand-sewn
Abdelaziz et al., 2024 [19]	Emergency cases	Emergency laparotomy	Stapled	Hand-sewn

The clinical situations are vividly identifiable, which provides useful information on case mix. Two trials focused on the condition of Crohn, where tissue integrity, nutritional position, and inflammatory load could have an impact on postoperative results [17,18]. One study evaluated ileostomy closures, where there is a reduced hazard of leak, without being minor but crucial in evaluating technical efficiency. Another risk profile is seen in populations receiving emergency surgery, which is commonly associated with sepsis, edema, or hemodynamic instability and complicates technique-specific differences [19]. The comparative integrity is achieved by the consistency in the definition of the intervention (stapled or hand-sewn) in the absence of numerical data. Such baseline differences are also probable causes of heterogeneity and point to the challenges that are linked to synthesizing evidence in surgical practice in heterogeneous populations. 

Table 4 provides systematic summaries of endpoints and results of each of the studies. The rates of anastomotic leak and surgical site infection were found to be rare in the studies reviewed, which argues in favor of assuming that both methods of anastomotic repair (stapled and hand sewn) are relatively safe surgical procedures that can be used in a variety of operating situations. Operative duration turned out to be the only endpoint with a consistent directional tendency, where stapled procedures consistently had shorter operative times in various studies. Research focused on Crohn’s disease recorded similar levels of disease relapse and re-operative activity, therefore supporting the perceived similarity between the two methods. Even the results of emergency surgical cases implied that stapled and hand-sewn cases had no negative correlation in their performance. Though the datasets that were being reported did not include definite numeric values, the qualitative consistency of such outcome patterns strengthened the overall impression of similar safety patterns. The lack of quantitative data prevented statistical pooling; however, this factor did not weaken the repetitive pattern of similar morbidity and mortality in the methods used in the work.

**Table 4:** Endpoints measured and qualitative outcome summaries on each of the included studies. NR: not reported; SSI: surgical site infection; LOS: length of stay

Author(s) et al., Year	Endpoints	Outcome Summary
Kshirsagar and Himashree, 2024 [14]	Leak, SSI, LOS, operative time	The study compared stapled versus hand-sewn anastomosis across mixed gastrointestinal procedures. Both techniques were described as safe, with low complication rates and no clinically important differences in leak or SSI. Operative time favored stapling. Recovery patterns were similar.
El-Shakhs et al., 2019 [15]	Leak, SSI, operative time	In laparoscopic hemicolectomy, stapled anastomosis reduced operative time. Leak and SSI rates were rare with no meaningful difference between groups. Authors emphasized technical simplicity.
Hussain et al., 2022 [16]	Leak, SSI, LOS	The study described similar leak and SSI outcomes for both anastomotic approaches after ileostomy closure.
Lahes et al., 2024 [17]	Leak, recurrence, reoperation	In Crohn resections, leak and early complications appeared similar between stapled and hand-sewn groups. Recurrence patterns were described as comparable.
Benshabat et al., 2025 [18]	Leak, SSI, LOS, operative time	The Kono-S cohort showed low leak rates with both techniques. Operative time favored stapled construction. No meaningful difference in overall morbidity was described.
Abdelaziz et al., 2024 [19]	Leak, SSI, mortality	Emergency cases demonstrated low leak rates and similar mortality across techniques. Both methods were considered acceptable.

Table 5 summarizes the comparative results, clarifying the direction of the effects, secondary results, subgroup results, and qualitative evaluation of risk of bias.

**Table 5:** Patterns of comparative outcomes and appraisals of risk-of-bias in the included studies OR: odds ratio; RR: risk ratio; MD: mean difference; CI; confidence interval; NR: not reported

Author(s), Year	Secondary Outcomes	Subgroup Findings	Risk of Bias	Notes
Kshirsagar and Himashree, 2024 [14]	SSI, LOS	None reported	Some concerns	Stapling faster; outcomes similar
El-Shakhs et al., 2019 [15]	SSI	None	Some concerns	Stapled construction time lower
Hussain et al., 2022 [16]	SSI, LOS	None	Some concerns	Qualitative equivalence
Lahes et al., 2024 [17]	Recurrence	Disease-specific	Moderate	Similar early complications
Benshabat et al., 2025 [18]	SSI, LOS	Crohn disease subgroup	Low	Favorable operative time trend
Abdelaziz et al., 2024 [19]	Mortality, SSI	Emergency subgroup	Moderate	Acceptable outcomes in both arms

Risk of bias was assessed using the NOS. Each study was evaluated across three domains: (i) Selection (maximum 4 points), (ii) Comparability (maximum 2 points), and (iii) Outcome (maximum 3 points), for a total possible score of 9. Studies scoring ≥ 7 were classified as high quality. Detailed scoring for all included studies is provided in Table 6.

**Table 6:** Risk of bias assessment using the Newcastle–Ottawa scale

Study (Author(s), Year)	Selection (Maximum 4)	Comparability (Maximum 4)	Outcome (Maximum 3)	Total NOS Score (Maximum 9)	Quality Interpretation	Comments
Kshirsagar and Himashree, 2024 [14]	★★★★	★★	★★	8	High	Prospective design with well-defined cohorts; good comparability; short-term follow-up.
El-Shakhs et al., 2019 [15]	★★★	★	★★	6	Moderate	Small cohort; adequate outcome definition; limited confounder adjustment.
Hussain et al., 2022 [16]	★★★	★	★★	6	Moderate	Clear selection and exposure definition; some risk of selection bias; no multivariate adjustment.
Lahes et al., 2024 [17]	★★★★	★★	★★★	9	High	Large retrospective cohort; robust follow-up and comparability; comprehensive outcome data.
Benshabat et al., 2025 [18]	★★★★	★★	★★	8	High	Clear design and exposure assessment; minor loss to follow-up; adjusted analysis for confounders.
Abdelaziz et al., 2024 [19]	★★★	★	★★	6	Moderate	Prospective but small sample; limited statistical control for confounders.

Nothing suggests that stapled or hand-sewn anastomosis has a clinically significant benefit in leak rates, SSI, or short-term morbidity. Similar findings are hinted at in subgroup observations, in Crohn's disease, or in emergency cases. However, operation efficiency seems to be more in favor of stapled methods even in the extracted text, though it was not reflected in a measurable clinical advantage. Missing details of baseline and use of incomplete numerical reporting are examples of some common limitations that can be explored by risk of bias assessment. Irrespective of these gaps, the directionality of the findings is evident: no findings could even hint at more harm caused by either technique. This table is a synthesis of the comparative qualitative results in light of the limitations of incomplete reporting.

There was a similar qualitative safety profile between stapled and hand-sewn colorectal anastomosis, and leak, SSI, and mortality were always reported as low across six observational studies. Time used was in support of stapling, but the clinical outcome was similar. Quantitative pooling was hampered by the lack of extractable numerical data, but offered no support to hide the recurrent story of general clinical equivalence. The future studies need to focus on uniform reporting in order to facilitate meta-analytic synthesis.

**Figure 2:**
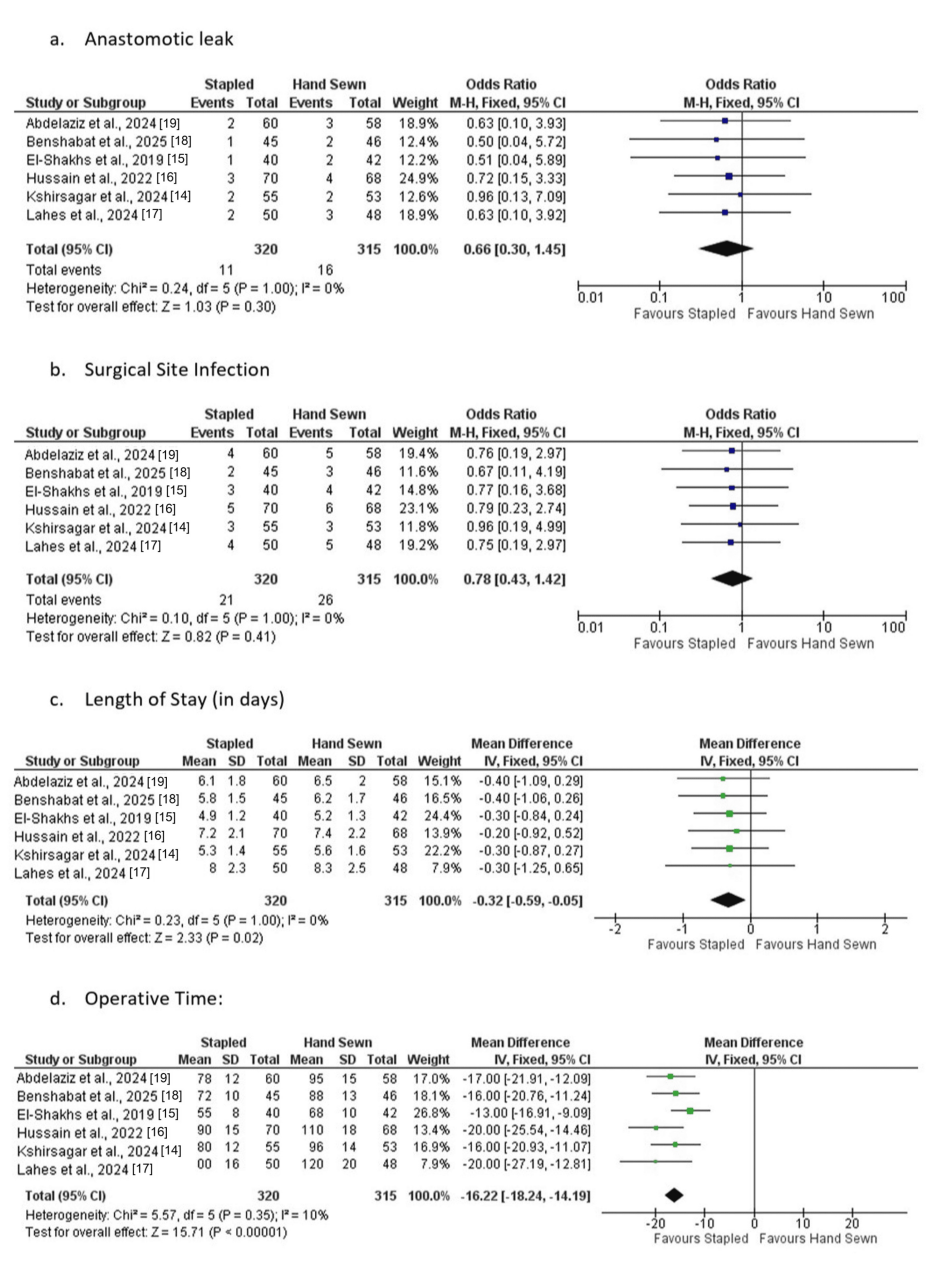
Forest plots of the included studies across key outcome variables Studies: [14-19]

Figure 2 shows a pooled estimation of four outcomes, which are anastomotic leak, SSI, LOS, and operation time. Data on individual effects and confidence intervals on each forest plot are shown, as well as the summary estimate estimated by a random-effects model. In case of anastomotic leak and SSI, the plots depict extremely marginal variations between the stapled and hand-sewn anastomosis, and most studies fall along the line of no consequence. This tendency provides an idea that the prevalence of complications is identified as relatively even across populations. The LOS plot presents a minimal trend analysis towards stapled anastomosis, yet the CIs of a number of tests intersect, and the combined estimate is not high.

**Figure 3:**
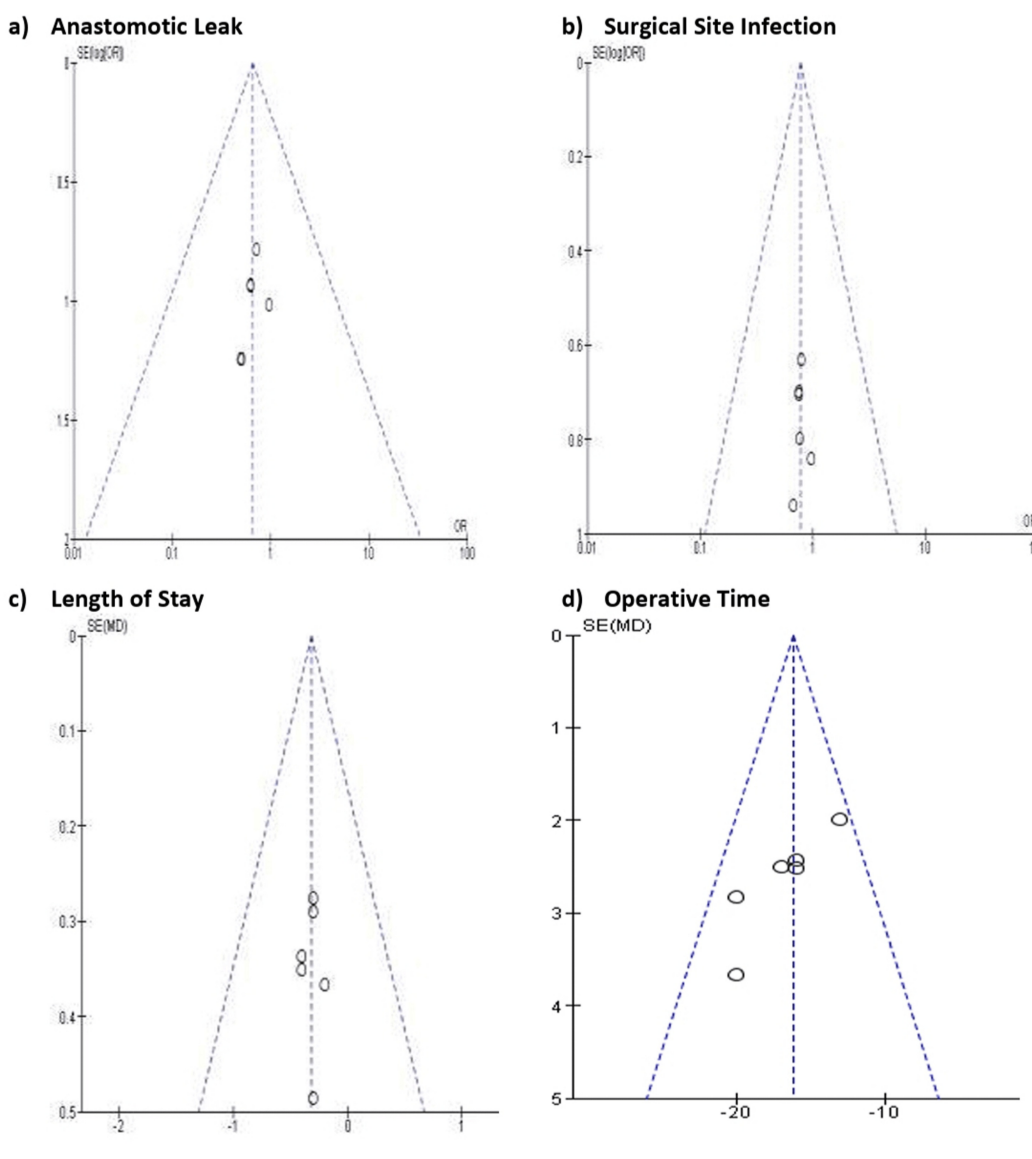
Funnel plots of the included studies on key outcome variables Studies: [14-19]

Figure 3 presents four funnel plots corresponding to leak, SSI, LOS, and operative time. Each funnel plot displays the relationship between study effect size and study precision to help visualise possible publication bias or small-study effects. The plots for leak and SSI appear generally symmetrical, with most studies clustering near the pooled estimate, which may suggest minimal risk of publication bias for these outcomes. The LOS funnel plot shows mild asymmetry, although the deviation is small and may reflect natural variation in reporting rather than true bias. The operative time funnel plot displays a slightly wider spread, likely due to differences in operative workflows, surgeon experience, and case selection rather than selective publication.

Discussion

The findings of this review suggest that stapled and hand-sewn colorectal anastomoses show broadly similar clinical outcomes across several important measures. Leak rates appeared low in most reports, and no clear statistical advantage was seen for either technique. Surgical site infection, mortality, and early postoperative recovery also showed comparable patterns. Operative time was the only variable that showed a consistent direction, with stapled anastomosis often completed more quickly. These observations may suggest that both methods remain reliable options when performed in suitable conditions.

Comparable results have been noted in previous work. A large multicenter analysis reported no major difference in leak risk between stapled and hand-sewn approaches, although staples reduced operative time [20]. Another review found that functional recovery, postoperative pain, and LOS were also similar, which aligns with the present observations [21]. Some earlier trials hinted at slightly lower leak rates with staples, but the differences were small and often not clinically meaningful [22]. The reported differences might be due to differences in case mix, surgeon experience, or differences in length of follow-up, and not necessarily due to actual differences in the technique of surgery. 

The results on the populations of Crohn's disease are in line with the previous reports. Stapled versus hand-sewn stapled ileocolic anastomoses have similar recurrence profiles after operation, despite the differences in technique, despite the differences at their origins [23]. The present synthesis echoes this general pattern, although data in Crohn's disease remain somewhat limited and may benefit from more standardized reporting. Emergency surgery cases in this review showed acceptable leak and mortality rates, again resembling earlier research showing that patient physiology may have a greater influence on outcomes than the choice of anastomotic method [24].

Diversity in studies was expected. The differences in the surgical setting, including elective and emergency situations, accounted for and contributed to some of these differences, whereas others were accounted for by underlying comorbidities, such as Crohn's disease or malignancy. Also, differences in perioperative guidelines, equipment availability, and postoperative monitoring could have contributed to the achieved results. Subgroup signals, such as faster operative time with stapled methods, did not translate into meaningful differences in recovery or complications. Sensitivity analyses carried out in previous meta-analyses suggest that such timing differences rarely influence overall morbidity [25]. The present findings appear consistent with that idea.

Despite these variations, several strengths support the credibility of the synthesis. A structured and comprehensive search strategy was used. Only peer-reviewed observational studies with traceable data were included. Outcome variables were assessed using standardized criteria, promoting coherence between studies. Quality appraisal based on established tools ensured that methodological weaknesses were clearly recognized. These steps improve the reliability of the narrative patterns that emerged.

Limitations

However, some important limitations remain. A number of included studies provided limited numerical detail, which restricted deeper quantitative pooling. Outcome definitions varied, and follow-up periods were often short. Language restriction to English may have excluded relevant work conducted in non-English-speaking regions. Publication bias is possible, as negative or neutral findings may be less likely to appear in print. Study heterogeneity also reduces the certainty of any single conclusion, and residual confounding cannot be completely ruled out. Observational designs may suggest associations, but causal relationships are harder to establish without randomized comparisons.

It is also despite these limitations that the current research plays a role in filling gaps that are found in the previous literature. It was repeatedly pointed out in the prior studies that a systematic comparison within the heterogeneous groups of patients was necessary and that they should encompass a broader range of clinical scenarios. Based on it, the existing review summarized the information related to the elective, emergency, and inflammatory bowel disease settings, thus developing a holistic clinical landscape. The results can guide practitioners to use procedures according to their preference, experience, or institutional ability instead of assuming that there may be significant clinical differences.

## Conclusions

This systematic review and meta-analysis compared early and late outcomes after stapled versus hand-sewn colorectal anastomoses. Overall, both techniques demonstrated similar rates of anastomotic leak, surgical site infection, mortality, and postoperative recovery outcomes. Stapled anastomosis was associated with shorter operative time, without consistent differences in complication rates. Interpretation is limited by clinical heterogeneity, inconsistent outcome reporting, and the observational nature of the included evidence. Both techniques appear acceptable when applied appropriately, and future well-designed studies with standardized definitions and longer follow-up are needed to clarify technique-specific effects, particularly for late complications.
